# Risk Factors for Nodal Failure in Patients with FIGO IIIC Cervical Cancer Receiving Definitive Image-Guided Radiotherapy

**DOI:** 10.3390/curroncol30120756

**Published:** 2023-12-08

**Authors:** Xiaoliang Liu, Xiaorong Hou, Ke Hu, Fuquan Zhang, Weiping Wang, Kang Ren

**Affiliations:** Department of Radiation Oncology, Peking Union Medical College Hospital, Chinese Academy of Medical Sciences & Peking Union Medical College, Beijing 100730, China; liuxiaoliang@pumch.cn (X.L.); zhangfq@pumch.cn (F.Z.); wangweiping@pumch.cn (W.W.); renkang@pumch.cn (K.R.)

**Keywords:** cervical cancer, definitive radiotherapy, nodal failure, nodal shrinkage, cycles of chemotherapy

## Abstract

Background: Nodal failure is a major failure pattern for patients with FIGO IIIC cervical cancer, which is further associated with worse survival. This study was designed to investigate risk factors for nodal failure in FIGO IIIC cervical cancer patients. Methods: The characteristics of positive lymph nodes (LNs) and relevant clinical factors of 162 FIGO IIIC cervical cancer patients were collected. The chi-square test and logistic regression model were used to identify risk factors for nodal failure. Results: In total, 368 positive LNs were identified, including 307 pelvic LNs and 61 para-aortic LNs. The nodal failure rates for all LNs, pelvic LNs, and para-aortic LNs were 9.2%, 7.8%, and 16.4%, respectively. After 20 fractions of RT, a nodal short diameter (D_20F_) ≥ 0.95 cm and a ratio of nodal shrinkage (ΔV_20F_) < 0.435 resulted; <4 cycles of chemotherapy indicated higher nodal failure rates for all LNs. For pelvic LNs, ΔV_20F_ < 0.435 and <4 cycles of chemotherapy were associated with a higher incidence of nodal failure. For para-aortic LNs, ΔV_20F_ < 0.435 was the only risk factor for nodal failure. Conclusions: Para-aortic LNs were more likely to experience nodal failure than pelvic LNs. Nodal shrinkage during radiotherapy and cycles of chemotherapy were associated with nodal failure in patients with FIGO IIIC cervical cancer.

## 1. Introduction

Lymph node metastasis (LNM) is an important adverse prognostic factor for patients with cervical cancer [1,2,3]. In our previous studies, the 3-year OS rates of cervical cancer patients with and without LNM were 69.3% and 88.4%, respectively [3]. In patients with para-aortic lymph node metastasis (PALNM), the 3-year OS rate decreased to 52.8% [4]. Nodal failure is also a major failure pattern in this group of patients. In the report by EMBRACE [5], the nodal failure rates for patients N+ before treatment were higher than those with N- (16% vs. 7%). Therefore, it is of great importance to explore the risk factors for nodal failure to improve the lymph node control rate and further improve the prognosis of cervical cancer patients with LNM.

According to the previous reports, the sizes of positive lymph nodes (LNs) [6], radiation dose [7], and SUV value of LNs in PET [8] are closely related to the effective control of LNs. A dose–volume effect was also observed regarding nodal control in a previous study [6]. However, few studies have focused on the response of lymph nodes to treatment, especially their early response, during radiotherapy [9]. Recently, a study from France revealed that the regression rate of primary cervical tumors during radiotherapy significantly affected local control and overall survival. For patients with a primary tumor regression rate of <90%, the local recurrence rate was 8.5 times higher than that in those with a regression rate of ≥90% [10]. Therefore, we speculate that the regression of positive LNs during radiotherapy may also be correlated with nodal control.

At our institute, all cervical cancer patients treated with intensity-modulated radiotherapy (IMRT) underwent re-CT simulation after 20 fractions of external beam radiotherapy (EBRT). We were able to collect lymph node imaging information after 20 EBRT fractions. In the present study, we collected information on positive LNs before and after 20 fractions of EBRT, as well as histology and other treatment factors, to explore the risk factors associated with nodal failure, especially the prognostic value of nodal shrinkage during radiotherapy, to provide a basis for the early adjustment of the treatment plan.

## 2. Materials and Methods

### 2.1. Patient Collection

Patients with cervical cancer, treated at Peking Union Medical College Hospital between November 2011 and December 2017, were retrospectively reviewed. The detailed inclusion criteria were as follows.

1. Biopsy-proven cervical cancer;
2. Imaging confirmed pelvic or para-aortic lymph node metastasis (FIGO 2018 IIIC);
3. Complete information on positive lymph nodes, including information before treatment and during follow-up:
  (1) For patients with pelvic LNM (PLNM), pelvic MRIs before and one month after treatment were required;
  (2) For patients with para-aortic LNM (PALNM), abdominal CTs before and one month after treatment were required;
4. Follow-up period exceeding six months;
5. No evidence of distant metastasis (DM) before the treatment;
6. Treatment with definitive radiotherapy.

### 2.2. Radiotherapy

All the enrolled patients were treated with definitive radiotherapy and concurrent chemotherapy. The clinical target volume (CTV) included the primary tumor, part of the vagina, cervix, uterus, parametrium, and pelvic lymph nodes (the common iliac, external iliac, internal iliac, obturator, and presacral lymph nodes). In patients with PALNM, the para-aortic region was also included in the CTV. Positive lymph nodes were defined as gross tumor volume (GTV_nd_). Lymph nodes with a short diameter ≥10 mm on CT or MR were regarded as metastatic. For patients receiving PET/CT, positive LNs were diagnosed by radiologists based on medical history, characteristics of LNs on CT, and value of SUVmax. A margin of 8–10 mm was added to the CTV to form the planning clinical target volume (PCTV) and the GTV_nd_ plus a 5 mm margin was defined as the planning gross tumor volume (PGTV_nd_). A dose of 50.4 Gy in 28 fractions (50.4 Gy/28 F) was prescribed to at least 95% of PCTV with IMRT. At least 95% of the PGTV_nd_ received about 60 Gy irradiation with the simultaneous integrated boost (SIB) technique.

Image-guided radiotherapy was conducted for all patients receiving IMRT at our institute. For patients treated with FF-IMRT or VMAT, weekly cone beam CT (CBCT) was administered. For patients receiving helical tomotherapy (HT), daily on-board megavoltage CT was routinely used.

All patients received 2-D or 3-D brachytherapy. The standard radiation dose to point A or the HR-CTV was 30 Gy in 5 fractions (30 Gy/5F). For patients with residual tumors after 5 fractions, another 6 Gy in one fraction was added.

All patients in the present study received regular follow-up examinations after treatment. Enhanced pelvic MRI and enhanced abdominal CT were performed one month after treatment. If the residual lymph nodes were still determined as metastatic by radiologists, then, an additional dose of 6 Gy in 3 fractions (95% PGTVnd) was prescribed to the LNs.

### 2.3. Chemotherapy

Concurrent chemotherapy was recommended for all patients without any contraindications. The first-line regimen was weekly cisplatin 40 mg/m^2^. Weekly paclitaxel 60–80 mg/m^2^ was an alternative treatment for patients with renal dysfunction.

### 2.4. Lymph Node Characteristics

The endpoint of this study was nodal failure, which was defined as follows: (1) Positive LNs achieved complete remission (CR) and then recurred during follow-up. (2) Positive LNs achieved partial remission (PR) or stable disease (SD) and then enlarged during follow-up. (3) The positive LNs showed progression (PD) during or after treatment. The definitions of CR, PR, SD, and PD were based on the RECIST 1.1.

**Figure 1:**
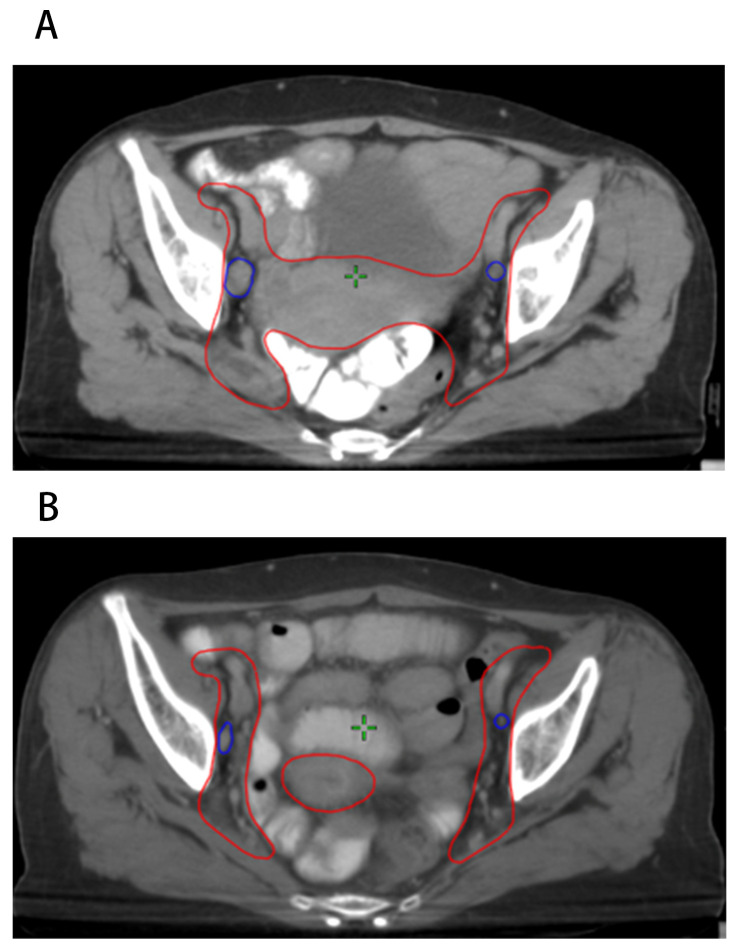
Positive pelvic LNs before and after 20 fractions of radiotherapy. (**A**) LNs before radiotherapy; (**B**) LNs after 20 fractions of radiotherapy.

As described above, patients with cervical cancer underwent re-CT simulation after 20 fractions of EBRT and a new EBRT plan was created. The LN characteristics collected included the short diameter before radiotherapy (D_pre_), volume before radiotherapy (V_pre_), short diameter after 20 fractions of EBRT (D_20F_), volume after 20 fractions of EBRT (V_20F_), short diameter regression rate after 20 fractions of EBRT (ΔD_20F_), and volume regression rate after 20 fractions of EBRT (ΔV_20F_). Figure 1 shows an example of patients with FIGO IIIC1 cervical cancer before and after 20 fractions of EBRT, which visualized the shrinkage of positive LNs during radiotherapy.

D_pre_, V_pre_, D_20F_, and V_20F_ were calculated based on CT simulation and re-CT simulation images (Eclipse System, Varian, Palo Alto, CA, USA). ΔD_20F_ was defined as (D_pre_ − D_20F_)/D_pre_, and ΔV_20F_ was defined as (V_pre_ − V_20F_)/V_pre_. Based on receiver operating characteristic (ROC) curves, the optimal cut-off values for D_pre_, V_pre_, D_20F_, V_20F_, ΔD_20F_, and ΔV_20F_ were 1.15 cm (Se 79.4%, Sp 41.9%), 2.85 cm^3^ (Se 82.4%, Sp 51.8%), 0.95 cm (Se 88.2%, Sp 64.4%), 3.45 cm^3^ (Se 64.7%, Sp 82.3%), 0.245 (Se 76.5%, Sp 75.4%), and 0.435 (Se 76.5%, Sp 75.2%), respectively (as Supplementary Figure S1A–F and Table 1 show).

The characteristics of the LNs (D_pre_, V_pre_, D_20F_, V_20F_, ΔD_20F_, and ΔV_20F_), histology, radiation dose of the LNs, and cycles of concurrent chemotherapy were selected as potential risk factors for nodal failure.

### 2.5. Statistics

Statistical analyses were performed using SPSS 23.0. Kaplan–Meier and log-rank tests were used to calculate and compare nodal failure rates between patients at different stages of disease. The chi-square test and logistic regression analysis were used to identify the risk factors for nodal failure. Receiver operating characteristic curves (ROC) were used to confirm the optimal cut-off values for continuous variables. Statistical differences were defined using a bilateral *p* < 0.05.

## 3. Results

### 3.1. Patients’ Characteristics

A total of 162 patients were enrolled with a median age of 50.5 years old (26–73 y). A total of 120 patients were classified as stage IIIC1 (FIGO 2018), whereas 42 patients were classified as stage IIIC2. Thirty-eight patients had positive pelvic and para-aortic lymph nodes and four patients had positive para-aortic lymph nodes only. Squamous cell carcinoma was the most prevalent type (of 150/162 92.6%). Adenocarcinoma, adenosquamous carcinoma, and other rare histological types were found in seven, three, and two patients, respectively. The median radiation dose to the positive lymph nodes was 60.2 Gy. Most patients received more than four cycles of concurrent chemotherapy (137/162, 84.6%). Table 2 shows the patients’ general conditions and treatment information.

### 3.2. Lymph Node Characteristics

A total of 368 lymph nodes were identified as positive, including 307 pelvic LNs and 61 para-aortic LNs. Most positive pelvic LNs were located in the external iliac area (201/307, 65.5%). Most positive PALNs were found in the left para-aortic region (41/61, 67.2%). The median D_pre_, V_pre_, D_20F_, V_20F_, ΔD_20F_, and ΔV_20F_ of the positive LNs were 1.2 cm, 3.0 cm^3^, 0.8 cm, 1.2 cm^3^, 0.33, and 0.60, respectively. In total, 276 LNs received doses of 60.2 Gy/28F, while 86 LNs (23.4%) received doses of less than 60.2 Gy, including 17 LNs with 56 Gy/28F (2 Gy/F), 49 LNs with 59.36 Gy/28F (2.12 Gy/F), and 20 LNs with 59.92 Gy/28F (2.14 Gy/F). These differences between dose prescriptions were due to different clinicians’ experiences. Six LNs were still identified as metastatic one month after treatment and they received an additional dose of 6 Gy in 3 fractions with a total dose of 66.2 Gy in 31 fractions. Table 3 shows the distribution and characteristics of the positive lymph nodes.

### 3.3. Nodal Failure

**Figure 2:**
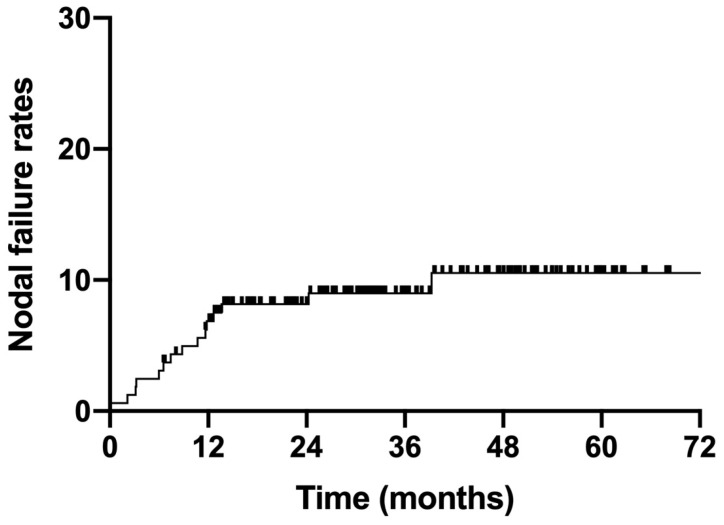
Nodal failure rates for all enrolled patients.

**Figure 3:**
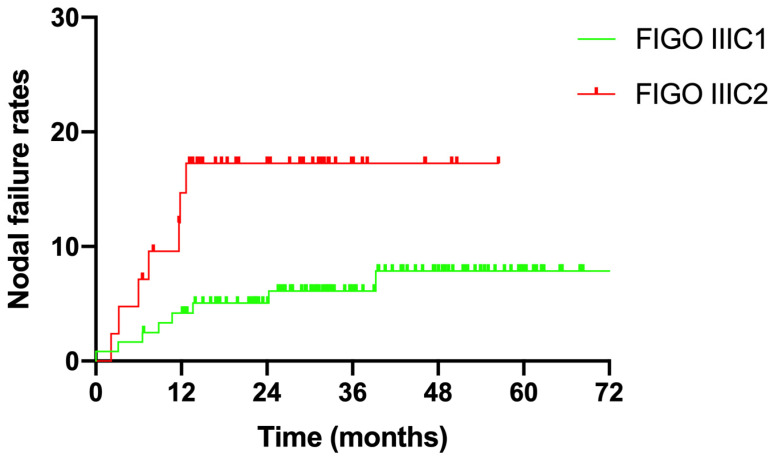
Nodal failure rates for different FIGO stages.

The median follow-up duration for all enrolled patients was 32.4 months (6.5–86.3 months). Fifteen patients experienced nodal failure, with a 3-year nodal failure rate of 9.0% (Figure 2). Eight patients with stage IIIC1 and seven patients with stage IIIC2 disease suffered nodal failure. The 3-year nodal failure rates for patients with FIGO stage IIIC1 and IIIC2 were 6.1% and 17.4%, respectively (Figure 3, *p* = 0.02).

The nodal failure rate was 9.2% (34/368) for all 368 positive LNs. A total of 24 of the 307 pelvic LNs and 10 of the 61 para-aortic LNs experienced nodal failure. The nodal failure rates for PLNs and PALNs were 7.8% and 16.4%, respectively (*p* = 0.035)

### 3.4. Risk Factors for Nodal Failure

As Table 4 shows, 368 LNs were positive. Univariate analysis showed that all nine risk factors were associated with nodal failure. Multivariate analysis identified that D_20F_ and ΔV_20F_ were significant risk factors for nodal failure. For lymph nodes with D_20F_ ≥ 0.95 cm and ΔV_20F_ < 0.435, the nodal failure rates were 6.8 times (*p* = 0.017) and 3.1 times (*p* = 0.045) higher as compared with those with D_20_ < 0.95 cm and ΔV_20F_ ≥ 0.435. Concurrent chemotherapy cycles were also related to nodal failure (<4 vs. ≥4 cycles, 22.8% vs. 5.5%, *p* = 0.006).

The 307 pelvic lymph nodes, D_pre_, V_pre_, D_20F_, V_20F_, ΔD_20F_, ΔV_20F_, and cycles of concurrent chemotherapy were associated with nodal failure according to univariate analysis. However, only ΔV_20F_ and cycles of chemotherapy significantly influenced nodal failure in the multivariate analysis. The nodal failure rates were 21.4% and 2.7% for LNs with ΔV20F < 0.435 and ≥0.435, respectively (*p* = 0.048). For LNs receiving <4 and ≥4 cycles of chemotherapy, the nodal failure rates were 20.7% and 4.9% (*p* = 0.001), respectively (Table 5).

For the 61 para-aortic lymph nodes, in the univariate analysis, D_20F_, V_20F_, ΔD_20F_, ΔV_20F_, and radiation dose significantly affected nodal failure, while only ΔV_20F_ remained an independent factor for nodal failure in the multivariate analysis. For LNs with ΔV_20F_ < 0.435, the nodal failure rate was 32.0%, approximately eleven times that for LNs with ΔV_20F_ ≥ 0.435 (32.0% vs. 5.6%, *p* = 0.015) (Table 6).

## 4. Discussion

Nodal failure is a major failure pattern in patients with cervical cancer, especially those with FIGO stage IIIC disease. According to data from EMBRACE, the nodal failure rate was 16% for patients with positive LNs before treatment, whereas it was only 7% for those with negative LNs [5]. Therefore, confirming risk factors for nodal failure in patients with FIGO IIIC cervical cancer might be a reasonable way to improve the prognosis in this group of patients. Our study first confirmed that nodal shrinkage after 20 fractions of EBRT (ΔV_20F_) was significantly correlated with the control of LNs. The nodal failure rates were 3.1% and 23.9% for LNs with ΔV_20F_ ≥ 0.435 and < 0.435, respectively (*p* < 0.001). In the stratified analysis, ΔV_20F_ effectively predicted nodal failure in both pelvic and para-aortic LNs. Additionally, the cut-off value of ΔV_20F_ was calculated based on ROC curves, with a high sensitivity of 76.5% and specificity of 75.2%. This methodology was quite scientific and reasonable. Our study design was quite similar to another study which revealed that regression of the primary cervical tumor during definitive CCRT significantly influenced local control and survival [10]. For patients with a GTV reduction of <90%, the local recurrence rate was 8.5 times than that for patients with a GTV reduction of ≥90% at the time of brachytherapy and the overall survival was 45.0% vs. 92.0% (*p* < 0.001). However, few studies have discussed the relationship between nodal shrinkage during treatment and nodal failure. Only one study from Japan [9] revealed that for LNs with diameters <10 mm and ≥10 mm after 50 Gy, the LN control rates were 96.7% and 75.7%, respectively (*p* < 0.001). Compared with the Japanese study [9], our study made an early prediction of nodal failure at the time of 40–43 Gy/20F, which could help clinicians adjust treatment plans for different patients in time.

In the present study, the characteristics of positive LNs before radiotherapy, such as nodal volume (V_pre_) and short diameter (D_pre_) of LNs, were related to nodal failure only according to univariate analysis. However, a previous study illustrated that the nodal volume before treatment was an independent factor for nodal failure [6]; for LNs with nodal volumes ≥ 3 cm^3^, the nodal failure rate was 8.2 times that for those with nodal volumes < 3 cm. Simultaneously, patients with large LNs (short diameter ≥ 15 mm) were more likely to experience local regional recurrence and distant metastasis [11]. Based on these reports, the pre-radiotherapy characteristics of LNs should not be ignored.

Radiation dose is another critical factor for nodal control. Wakatsuki et al. reported that in 16 LNs with ≤58 Gy, 9 LNs experienced recurrence. However, nodal failure did not occur in the other 21 LNs that were treated with >58 Gy [9]. Kim Yj treated cervical cancer patients with helical tomotherapy and the median radiation dose was 62.6 Gy (EQD2 53.3–77.9 Gy) for 58 LNs. During a long-term follow-up, 52 LNs maintained a complete response (CR) and only 3 LNs developed recurrence [7]. Bacorro et al. explored the dose–volume effect of LNs [6] and confirmed that a dose of >60 Gy was imperative for larger LNs (>2 cm). Additionally, they found that the SIB technique may provide potential benefits for nodal control. At our institute, the standard dose for positive LNs is 60.2 Gy in 28 fractions and we use the SIB technique in most cases. An additional dose was prescribed for some residual LNs. In the present study, 86 LNs received doses of less than 60 Gy, while the other 282 LNs received >60 Gy irradiation. The nodal failure rates for these two groups were 18.4% and 6.4%, respectively (*p* = 0.001). But the effect of radiation dose on nodal failure was counteracted by other factors according to multivariate analysis. Since the radiation dose was almost the same for all LNs, only five dose segmentation models existed (56 Gy/20 F, 59.36 Gy/28 F, 59.92 Gy/28 F, 60.2 Gy/28 F, and 66.2 Gy/31 F) and we were unable to determine a dose–volume effect regarding nodal failure. Based on our treatment model, the overall nodal failure rate was 9.2% for 368 lymph nodes and 7.8% for 307 pelvic lymph nodes, which was not inferior to that reported in previous studies [5].

The relationship between the number of concurrent chemotherapy cycles and prognosis in patients with cervical cancer has been reported in previous studies. In patients with FIGO IIIC1 cervical cancer, five or more cycles of chemotherapy can decrease the distant metastasis rate [12]. Another study from our department indicated that four or more cycles of concurrent chemotherapy could improve survival in FIGO IIIC1 cervical cancer patients; for patients receiving 0, 1–3 and ≥4 cycles of concurrent chemotherapy, the 3-year OS were 69.4%, 70.4%, and 84.4% (*p* = 0.009), respectively [13]. The present study further confirmed the relationship between the number of chemotherapy cycles and LN control. The nodal failure rates for 0–3 cycles and ≥4 cycles were 22.8% and 5.5%, respectively (*p* < 0.001). In other words, our study showed that at least four cycles of concurrent chemotherapy should be prescribed for high-risk cervical cancer patients.

Another important finding of this study was that the nodal failure rate of para-aortic LNs was higher than that of pelvic LNs (16.4% vs. 7.8%, *p* = 0.035). There are three possible reasons for this finding. First, most of the enrolled FIGO IIIC2 patients had pelvic LNM simultaneously (38/42, 90.5%), indicating that this group of patients had a high tumor burden. The intensity of conventional CCRT may not be sufficient for tumor control and other adjuvant therapies may be of potential value [14]. Second, the toxicity of the duodenum is an important issue when performing abdominal radiotherapy; the reported 3-year duodenal toxicity was 11.7% [15] and toxicity was also related to V55 of the duodenum [14,16]. If the positive LNs are anatomically closely related to the duodenum, the dose to the positive LNs may be sacrificed to protect the duodenum, which would further impair LN control. Finally, brachytherapy resulted in an additional dose to the pelvic LNs. The reported 2-Gy equivalent doses D98 for the obturator, internal iliac, external iliac, and common iliac were 4.4 ± 1.9 Gy, 5.4 ± 1.3 Gy, 4.3 ± 2.1 Gy, and 2.8 ± 2.5 Gy, respectively. However, no doses were delivered to the para-aortic LNs with brachytherapy [17]. Additional doses may be associated with the relevant high control rate of pelvic LNs.

Although the present study confirmed the relationship between nodal shrinkage during definitive radiotherapy and nodal failure in locally advanced cervical cancer, it has several limitations. First, this was a retrospective study; some patients lacked complete data on LN evaluation and only 162 patients were enrolled, which might have resulted in selection bias. Moreover, as mentioned above, only five dose segmentation models existed in the present study (56 Gy/20F, 59.36 Gy/28F, 59.92 Gy/28F, 60.2 Gy/28F, and 66.2 Gy/31F); almost all enrolled LNs received about 60 Gy irradiation. The dose consistency was high. Therefore, we could not conclude a dose–volume effect regarding LN control at present.

## 5. Conclusions

Compared to pelvic LNs, para-aortic LNs are more likely to experience nodal failure. Nodal shrinkage during definitive radiotherapy significantly affects nodal control in patients with locally advanced cervical cancer. ΔV_20F_ < 0.435 in LNs indicated a higher nodal failure rate. Four or more cycles of chemotherapy can improve the LN control rate in patients with FIGO IIIC cervical cancer.

## Figures and Tables

**Table 1:** Optimal cut-off values of LN characteristics for predicting nodal failure.

Characteristics	Cut-Off Value	Sensitivity	Specificity	AUC 95%CI	*p* Value
D_pre_	1.15 cm	79.4%	41.9%	0.65 (0.55–0.74)	0.005
V_pre_	2.85 cm^3^	82.4%	51.8%	0.70 (0.60–0.80)	<0.001
D_20F_	0.95 cm	88.2%	64.4%	0.78 (0.70–0.86)	<0.001
V_20F_	3.45 cm^3^	64.7%	82.3%	0.79 (0.72–0.87)	<0.001
ΔD_20F_	0.245	76.5%	75.4%	0.78 (0.68–0.88)	<0.001
ΔV_20F_	0.435	76.5%	75.2%	0.77 (0.67–0.87)	<0.001

Notes: D_pre_ = nodal short diameter before radiotherapy; V_pre_ = nodal volume before radiotherapy; D_20F_ = nodal short diameter after 20 fractions of EBRT; V_20F_ = nodal volume after 20 fractions of EBRT; ΔD_20F_ = short diameter regression rate after 20 fractions of EBRT; ΔV_20F_ = volume regression rate after 20 fractions of EBRT.

**Table 2:** Patients’ characteristics.

Characteristics	N. (%)
Age	
Median: 50.5 years (26–73 years)	
Histology	
Squamous cell carcinoma	150 (92.6%)
Adenocarcinoma	7 (4.3%)
Adenosquamous carcinoma	3 (1.9%)
Others	2 (1.2%)
Lymph node metastasis	
PLNM only	120 (74.1%)
PALNM only	4 (2.5%)
PLN + PALNM	38 (23.4%)
FIGO stage (2018)	
IIIC1	120 (74.1%)
IIIC2	42 (25.9%)
Radiation dose of LNM	
Median: 60.2 Gy	
≥60 Gy	121 (74.7%)
<60 Gy	41 (25.3%)
Cycles of concurrent chemotherapy	
0	8 (4.9%)
1–3	17 (10.5%)
≥4	137 (84.6%)
Total	162 (100%)

Notes: PLNM = pelvic lymph node metastasis; PALNM = para-aortic lymph node metastasis.

**Table 3:** Lymph node characteristics.

Characteristics	N. (%)
Distribution	
Positive pelvic LN	307 (83.4%)
Common iliac region	46 (15.0%)
External iliac region	201 (65.5%)
Internal iliac region	18 (5.8%)
Obturator region	38 (12.4%)
Presacral region	1 (0.3%)
Perirectal region	3 (1.0%)
Positive para-aortic LN	61 (16.6%)
LPA	41 (67.2%)
AC	19 (31.2%)
RPC	1 (1.6%)
D_pre_ (cm)	
Median 1.2 (1.0–3.4)	
<1.15	147 (39.9%)
≥1.15	221 (60.1%)
V_pre_ (cm^3^)	
Median 3.0 (1.0–54.1)	
<2.85	179 (48.6%)
≥2.85	189 (51.4%)
D_20F_ (cm)	
Median 0.8 (0.2–2.7)	
<0.95	219 (59.5%)
≥0.95	149 (40.5%)
V_20F_ (cm^3^)	
Median 1.2 (0.1–38.5)	
<3.45	287 (78.0%)
≥3.45	81 (22.0%)
ΔD_20F_	
Median 0.36 (−0.67–0.80)	
≥0.245	260 (70.7%)
<0.245	108 (29.3%)
ΔV_20F_	
Median 0.61 (−2.03–0.95)	
≥0.435	259 (70.4%)
<0.435	109 (29.6%)
Radiation dose (95% PGTVnd)	
56 Gy/28 F	17 (4.6%)
59.36 Gy/28 F	49 (13.3%)
59.92 Gy/28 F	20 (5.5%)
60.2 Gy/28 F	276 (75.0%)
66.2 Gy/31 F	6 (1.6%)
Total	368 (100%)

Notes: LPA = regions between aorta and left psoas muscle; AC = regions between inferior vena cava and aorta; RPC = regions between inferior vena cava and right psoas muscle.

**Table 4:** Univariate and multivariate analyses of nodal failure for all enrolled LNs.

Risk Factors	Nodal Failure Rate	Univariate Analysis (χ2 Test)	Multivariate Analysis (Logistic Test)	
		*p* Value	HR (95%CI)	*p* Value
D_pre_ (cm)				
≥1.15 vs. <1.15	12.2% vs. 4.8%	0.016	—	—
V_pre_ (cm^3^)				
≥2.85 vs. <2.85	14.8% vs. 3.4%	<0.001	—	—
D_20F_ (cm)				
≥0.95 vs. <0.95	20.1% vs. 1.8%	<0.001	6.855 (1.402–33.518)	0.017
V_20F_ (cm^3^)				
≥3.45 vs. <3.45	27.2% vs. 4.2%	<0.001	—	—
ΔD_20F_				
<0.245 vs. ≥0.245	24.1% vs. 3.1%	<0.001	—	—
ΔV_20F_				
<0.435 vs. ≥0.435	23.9% vs. 3.1%	<0.001	3.069 (1.027–9.170)	0.045
Histology				
Non-SCC vs. SCC	20.0% vs. 8.3%	0.034	—	—
Radiation dose (Gy)				
<60 vs. ≥60	18.4% vs. 6.4%	0.001	—	—
Cycles of CT				
<4 vs. ≥4	22.8% vs. 5.5%	<0.001	3.635 (1.441–9.166)	0.006
Distribution of LNs				
PALNM vs. PLNM	16.4% vs. 7.8%	0.035	—	—

**Table 5:** Univariate and multivariate analyses of nodal failure for pelvic LNs.

Risk Factors	Nodal Failure Rate	Univariate Analysis (χ2 Test)	Multivariate Analysis (Logistic Test)	
		*p* Value	HR (95%CI)	*p* Value
D_pre_ (cm)				
≥1.15 vs. <1.15	10.9% vs. 2.6%	0.009	—	—
V_pre_ (cm^3^)				
≥2.85 vs. <2.85	13.6% vs. 2.0%	<0.001	—	—
D_20F_ (cm)				
≥0.95 vs. <0.95	16.2% vs. 2.2%	<0.001	—	—
V_20F_ (cm^3^)				
≥3.45 vs. <3.45	23.4% vs. 3.7%	<0.001	—	—
ΔD_20F_				
<0.245 vs. ≥0.245	21.7% vs. 2.7%	<0.001	—	—
ΔV_20F_				
<0.435 vs. ≥0.435	21.4% vs. 2.7%	<0.001	3.737 (1.009–13.845)	0.048
Histology				
Non-SCC vs. SCC	16.7% vs. 7.1%	0.093	—	—
Radiation dose (Gy)				
<60 vs. ≥60	12.7% vs. 6.4%	0.082	—	—
Cycles of CT				
<4 vs. ≥4	20.7% vs. 4.9%	<0.001	5.087 (1.897–13.641)	0.001

**Table 6:** Univariate and multivariate analyses of nodal failure for para-aortic LNs.

Risk Factors	Nodal Failure Rate	Univariate Analysis (χ2 Test)	Multivariate Analysis (Logistic Test)	
		*p* Value	HR (95%CI)	*p* Value
D_pre_ (cm)				
≥1.15 vs. <1.15	20.7% vs. 12.5%	0.388	—	—
V_pre_ (cm^3^)				
≥2.85 vs. <2.85	20.0% vs. 11.5%	0.377	—	—
D_20F_ (cm)				
≥0.95 vs. <0.95	40.0% vs. 0	<0.001	—	—
V_20F_ (cm^3^)				
≥3.45 vs. <3.45	41.2% vs. 6.8%	0.001	—	—
ΔD_20F_				
<0.245 vs. ≥0.245	32.0% vs. 5.6%	0.006	—	—
ΔV_20F_				
<0.435 vs. ≥0.435	32.0% vs. 5.6%	0.006	11.000 (1.603–75.502)	0.015
Histology				
Non-SCC vs. SCC	33.3% vs. 14.5%	0.238	—	—
Radiation dose (Gy)				
<60 vs. ≥60	43.8% vs. 6.7%	0.001	—	—
Cycles of CT				
<4 vs. ≥4	28.6% vs. 10.0%	0.063	—	—

## Data Availability

The data presented in this study are available upon request from the corresponding author. The data are not publicly available due to privacy reasons.

## Supplementary Materials

The following supporting information can be downloaded at: https://www.mdpi.com/article/10.3390/curroncol30120756/s1, Figure S1: ROC curves for characteristics of LNs predicting nodal failure. (A) ROC curve for Dpre predicting nodal failure; (B) ROC curve for Vpre predicting nodal failure; (C) ROC curve for D20 predicting nodal failure; (D) ROC curve for V20 predicting nodal failure; (E) ROC curve for ΔD20F predicting nodal failure; (F) ROC curve for ΔV20F predicting nodal failure.
