# Supplementary material for: Risk Factors for Nodal Failure in Patients with FIGO IIIC Cervical Cancer Receiving Definitive Image-Guided Radiotherapy
Source: Curr Oncol. 2023 Dec 8;30(12):10385–95. doi: 10.3390/curroncol30120756 (PMC10742234; doi:10.3390/curroncol30120756)
Supplement: Supplementary file 1 [file curroncol-30-00756-s001.zip › curroncol-2720218-supplementary.pdf]

Current Oncology

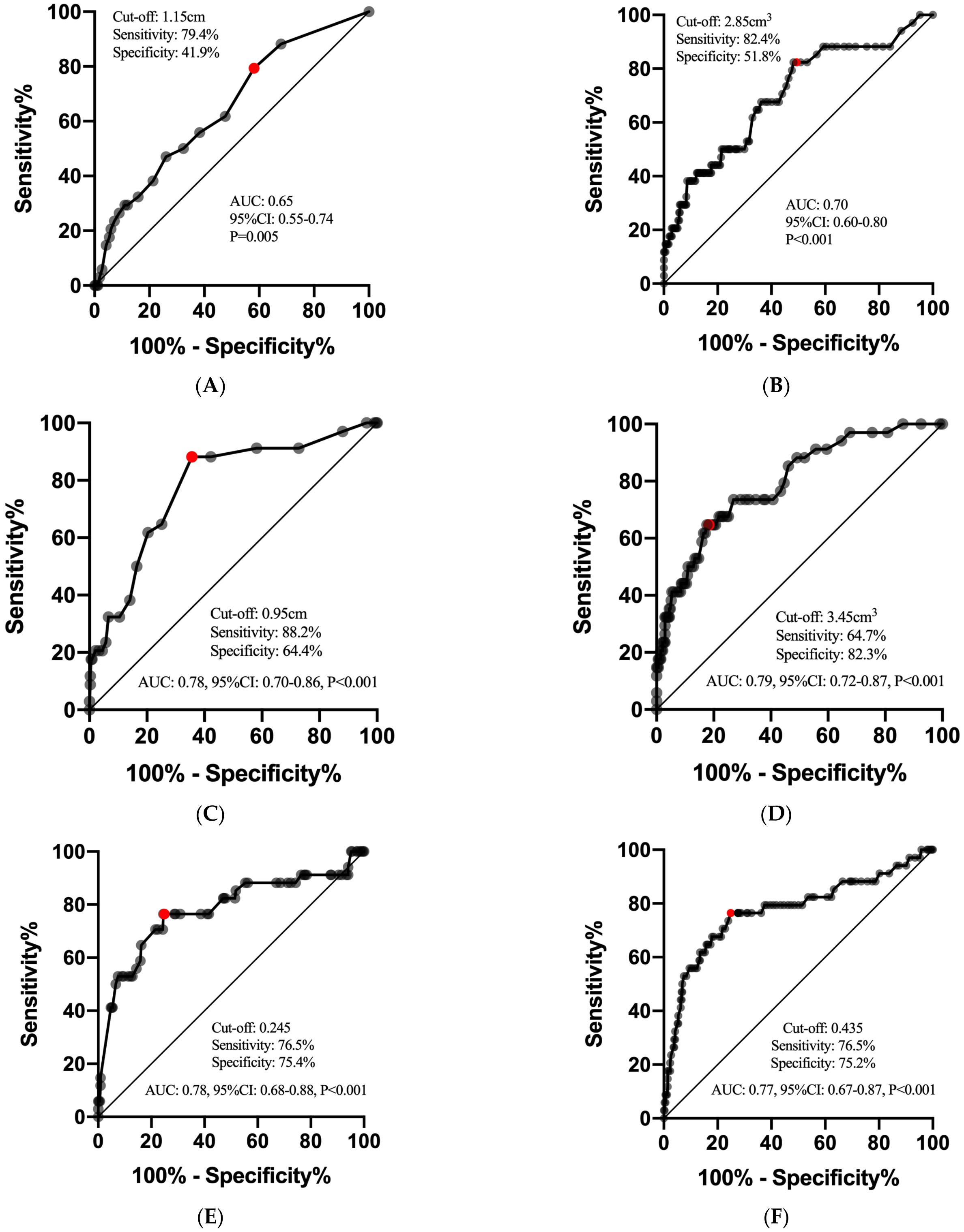

Figure S1. ROC curves for characteristics of LNs predicting nodal failure. (**A**) ROC curve for D_{pre} predicting nodal failure. The red point referred to the optimal cut-off value for D_{pre} (1.15cm) (**B**). ROC curve for V_{pre} predicting nodal failure. The red point referred to the optimal cut-off value for V_{pre} (2.85cm^3); (**C**) ROC curve for D_{20} predicting nodal failure. The red point referred to the optimal cut-off value for D_{20} (0.95cm); (**D**) ROC curve for V_{20} predicting nodal failure. The red point referred to the optimal cut-off value for V_{20} (3.45cm^3); (**E**) ROC curve for ΔD_{20F} predicting nodal failure. The red point referred to the optimal cut-off value forΔD_{20F} (0.245); (**F**) ROC curve for ΔV_{20F} predicting nodal failure. The red point referred to the optimal cut-off value forΔV_{20F} (0.435).
